# Pharmacy Compounded Medicines for Patients With Rare Diseases: Lessons Learned From Chenodeoxycholic Acid and Cholic Acid

**DOI:** 10.3389/fphar.2021.758210

**Published:** 2021-09-28

**Authors:** Yasmin Polak, Bart A. W. Jacobs, E. Marleen Kemper

**Affiliations:** ^1^ Department of Pharmacy and Clinical Pharmacology, University of Amsterdam, Amsterdam, Netherlands; ^2^ Platform Medicine for Society, Amsterdam, Netherlands

**Keywords:** pharmacy compounding, rare diseases, chenodeoxycholic acid, cholic acid, orphan medicines, bile acid synthesis defects, cerebrotendinous xanthomathosis

## Abstract

Patients with rare diseases are often confronted with the fact that effective medicines are unavailable or simply not being developed. This situation jeopardizes the health of a large population of vulnerable patients with rare diseases. Pharmacy compounded formulations can provide a safe alternative when authorized treatments are unavailable or unsuitable. Practical guidelines on how to develop and implement pharmacy compounded formulations for patients with rare diseases are limited. The aim of this article is to provide guidance for when and how to apply pharmacy compounded formulations for patients with rare diseases. This is illustrated with two challenging examples: the development and implementation of pharmacy compounding of 1) chenodeoxycholic acid (CDCA) capsules for patients with cerebrotendinous xanthomatosis (CTX) and 2) cholic acid (CA) capsules for patients with rare bile acid synthesis defects (BASD). All critical steps of the development of CDCA and CA capsules are explained and summarized in a practical guideline.

## Introduction

Patients with rare diseases are often confronted with the fact that effective pharmacotherapeutic treatments are unavailable. For some (ultra) rare diseases, medicines are simply not developed because the number of eligible patients is too small for a financially beneficial product (Joint evaluation of Regulation (EC), 2020). This problem jeopardizes the health of patients with rare diseases. Pharmacy compounded formulations can provide an alternative route for effective and safe pharmacotherapy when authorized orphan medicines are unavailable or unsuitable (Dooms and Cavalho, 2018).

Pharmacists are healthcare professionals with knowledge on medicine compounding and by European law they are certified to compound medicines for patients with a medical need. Although pharmacy compounded formulations are often perceived as cheaper “copies” of, or of lesser quality than authorized medicines, they do have to comply with strict laws and regulations to ensure product quality and patient safety. Unfortunately, practical guidelines on how to implement pharmacy compounded formulations for patients with rare diseases are limited.

**FIGURE 1:**
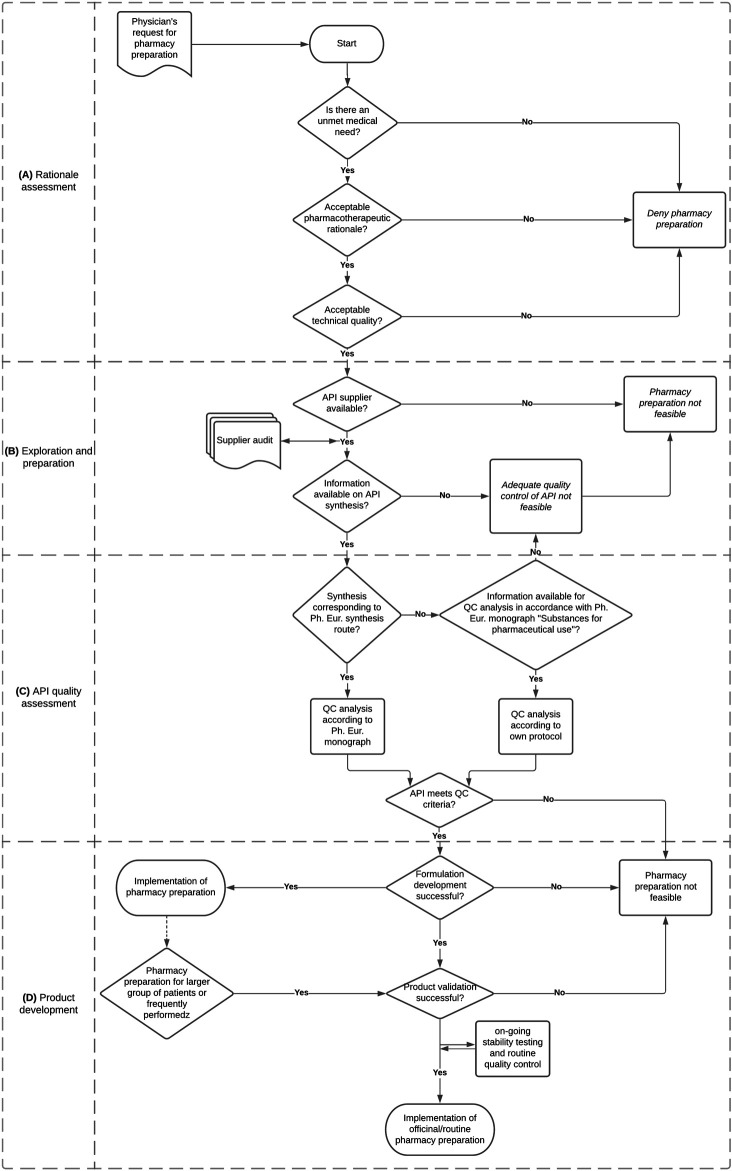
Flowchart for the development and implementation of a pharmacy compounded formulation.

In this article we share our experiences and perspectives to provide practical guidance on the critical steps for the development and implementation of pharmacy compounded formulations for patients with rare diseases. The critical steps are illustrated by two cases we worked on in the past years: pharmacy compounding of chenodeoxycholic acid (CDCA) capsules for patients with cerebrotendinous xanthomatosis (CTX) and cholic acid (CA) capsules for patients with rare bile acid synthesis defects (BASD). For both products the assessments and conclusions for each critical steps are summarized in Table 1. Based on our experiences, we developed a flowchart to provide practical guidance for the development and implementation of pharmacy compounded medicines using starting materials (Figure 1).

**TABLE 1:** Explanation and implementation of critical steps in the development process of pharmacy compounded CDCA and CA capsules.

Step	Chenodeoxycholic acid		Cholic acid		
Pharmacotherapeutic rationale	The efficacy and safety of CDCA for the treatment of CTX has been demonstrated by multiple studies since the seventies (Salen and Steiner, 2017; Berginer et al., 1989; Salen et al., 1991; Batta et al., 1985; Stelten et al., 2019; Verrips et al., 2020), EMA (2016)		Paucity of clinical evidence for the efficacy of CA treatment of BASDs or ZSD. EU authorization was based on case reports of confirmed or suspected 3β-HSD (n = 21) and Δ4-3-oxoR (n = 7) patients treated with CA (Laboratoires CTRS, 2013), Klouwer (2019)		
Setting	Standard care		Clinical trial		
Number of patients	60		20		
Sourcing of API and supplier audit	API supplier found in the Netherlands		API supplier found in the Netherlands		
	GMP API manufacturer resides outside the EU.		GMP API manufacturer resides outside the EU.		
	The manufacturer was audited on site by a subcontracted third party. The manufacturer was willing to share the DMF, which gave full insight into the API synthesis route and allowed for adequate API quality assessment		The manufacturer had recently been audited on GMP and shared the audit report. The manufacturer was not willing to share the DMF, but could provide the necessary information, data and statements to allow for adequate API quality assessment		
API quality control	API synthesis route is comparable to Ph. Eur. synthesis route with no critical deviations. API QC-analysis was performed according to general Ph. Eur. monograph *Substances for Pharmaceutical Use* (Ph.Eur., 2018b) and specific Ph. Eur. substance monograph *Chenodeoxycholic Acid* (Ph.Eur., 2017a). A subcontracting GMP QC laboratory performs EU QC-analysis on each batch. In-house identity testing through infrared spectroscopy on each container		No specific Ph. Eur. synthesis route or monograph available		
			API specifications were determined based on the synthesis route and general Ph. Eur. monograph Substances for Pharmaceutical Use (Ph.Eur., 2018b), and specific Ph. Eur. substance monographs of related substances *Chenodeoxycholic Acid* (Ph.Eur., 2017a) and *Ursodeoxycholic Acid* (Ph.Eur., 2017b). A subcontracting GMP QC laboratory performs EU QC-analysis on each batch. In-house identity testing through infrared spectroscopy on each container		
Formulation development	BCS classification: II		BCS classification: II		
	Broad therapeutic window, dose titration based on serum biochemical profile and liver transaminases levels. (In)compatibility with known excipients: N/A		Broad therapeutic window, dose titration based on serum biochemical profile, liver transaminases levels and side effects (In)compatibility with known excipients: N/A		
	Capsules formulation CDCA-Leadiant^®^ 250 mg (Leadiant, 2017) Pharmacy compounded CDCA 25–250 mg	Chenodeoxycholic acid	Capsules formulation	Cholic acid	
		Maize starch	Orphacol^®^ 50 and 250 mg (Laboratoires CTRS, 2013)	Lactose monohydrate	
		Colloidal anhydrous silica	Pharmacy compounded CA	Colloidal anhydrous silica
		Magnesium stearate	25–250 mg	Magnesium stearate
		Water		Size 3 and 0 hard gelatin capsule
		Size 0 hard gelatin capsule		Cholic acid
		Chenodeoxycholic acid		Lactose monohydrate
		Lactose monohydrate		Colloidal anhydrous silica
		Colloidal anhydrous silica		Size 3–0 hard gelatin capsule
		Size 3–0 hard gelatin capsule		
Batch size	100–400 capsules		200–1,200 capsules		
Product validation	No initial product validation required		Initial product validation required and performed on worst and best case formulation (n = 3)		
	Product validation required due to the amount of batches compounded per year (n > 50)		Tests: appearance, identity, LOD, content, content uniformity, related substances, microbiological quality, dissolution- and disintegration rate		
	Tests: appearance, identity, LOD, content, content uniformity, related substances, microbiological quality, dissolution- and disintegration rate				
Stability	6 months (15–25°C)		3 months (15–25°C)		
	Based on API- and excipients properties described in literature		On-going stability study performed on worst and best case formulation (n = 3) under normal conditions (25 ± 2°C/60 ± 5%RH) and stressed conditions (40 ± 2°C/75 ± 5%RH)		
Medicinal product quality control	QC analysis (n = 10) on appearance, average weight (deviates ≤3.0% from theoretical weight), uniformity of mass (RSD ≤4.0%) and homogeneity (indicated by average weight and uniformity)		QC analysis (n = 10) on identity, appearance, average weight (deviates ≤3.0% from theoretical weight), uniformity of mass (RSD ≤4.0%) and homogeneity (indicated by average weight and uniformity)		
	Performed during production process		Performed after production process by independent QC laboratory		

N/A = not applicable, DMF = Drug Master File, QC = Quality Control, GMP = Good Manufacturing Practice, EU = European union, LOD = Loss on Drying, RSD = Relative Standard Deviation, RH = Relative Humidity, BASD = bile acid synthesis defect, ZSD = Zellweger spectrum disorders, API = Active Pharmaceutical Ingredient, CTX = Cerebrotendinous Xanthomatosis, CDCA = chenodeoxycholic acid, CA = cholic acid, BCS = Biopharmaceutics Classification System, 3β-HSD = 3β-hydroxy-Δ5-C27-steroid oxidoreductase, Δ4-3-oxoR = Δ4-3-oxosteroid-5β-reductase, Ph. Eur. = European Pharmacopoeia

### Background of Cases: CDCA and CA

CDCA capsules were originally developed decades ago for the treatment of gallstones (Iser and Sali, 1981; Schoenfield and Lachin, 1981). It was prescribed off-label for the treatment of CTX since 1975 (Dutch National Health Care Institute, 2018). In 2017 CDCA was reintroduced in Europe (EU) as an authorized orphan medicine for the treatment of CTX (EMA, 2016; Leadiant, 2017). Although the reintroduction was not accompanied with significant pharmaceutical enhancement of the “old” formulation or extensive new research, the price increased 500 fold (Dutch National Health Care Institute, 2018). As a consequence, Dutch CTX patients were confronted with the fact that the treatment was no longer eligible for reimbursement and their treatment no longer accessible. In order to ensure accessibility of effective treatment for our own patients, we developed a pharmacy compounded formulation for CDCA capsules for Dutch CTX patients.

CA is an EU authorized treatment for BASDs due to in 3β-hydroxy-Δ5-C27-steroid oxidoreductase (3β-HSD) and Δ4-3-oxosteroid-5β-reductase (5β-reductase) deficiency (Laboratoires CTRS, 2013). However, in the Netherlands CA treatment is not considered part of standard care yet due to limited clinical evidence (Table 1). In order to study the long-term safety and efficacy, we developed CA capsules for BASD patients who participate in a clinical trial (Netherlands Trial Register, 2018, Trial NL8630).

## Legal Framework

There is worldwide no uniform legislation for compounding by pharmacists. For clarification, in the EU each member state formulates its own legislation for pharmacy compounding. To ensure safe and effective treatment of patients, pharmacy compounded medicines have to comply with high quality standards that are in line with national guidelines based on EU Directive 2001/83 (Directive 2001/83/EG). Therefore, pharmacists have to verify whether active ingredients and excipients comply with the European Pharmacopoeia (Ph. Eur.) and that the production facilities, qualification of personnel and documentation are in compliance with EU Directive 2001/83 (Directive 2001/83/EG). Besides the compliance of the quality control (QC) of starting materials, also the quality of the medicinal product has to comply with the specifications of the Ph. Eur. As the CA and CDCA were developed and made in the Netherlands, we worked according to the Dutch law (Dutch Medicines Act Decree).

## Pharmacotherapeutic Rationale for Pharmacy Compounding

In case a patient is likely to benefit from a pharmacotherapeutic intervention, but authorized medicines are unavailable, there is a so-called “unmet medical need”. In this situation the physician can explore other routes, e.g., *off-label* prescribing of an authorized medicine. When off-label use is not feasible, the physician can request a pharmacist to compound the specific medicine.

The decision to develop a pharmacy compounded formulation is preceded by a risk-benefit assessment (Resolution CM/Res, (2016), KNMP, 2016). First, the pharmacist assesses whether the requested pharmacotherapy has an added value for the patient based on expected treatment efficacy and safety, as well as the technical quality of the compounded formulation (Figure 1A). Eventually, the possibilities and uncertainties should be discussed with the prescribing physician and the patient.

### Pharmacotherapeutic Rationale for CDCA Capsules

The efficacy and safety of CDCA treatment in CTX patients have been demonstrated by multiple studies since the seventies (Table 1). After years of off-label treatment, CDCA treatment finally got EU authorization for the treatment of CTX in 2017 (EMA, 2016; Leadiant, 2017). However, the EU authorized medicine Chenodeoxycholic acid Leadiant^®^ has not been accessible for Dutch patients since April 2018 because it has been rejected for reimbursement by the Dutch health insurance due to its inexplicable high price (Dutch National Health Care Institute, 2018). This resulted in an unmet medical need for CTX patients, which could be solved by pharmacy compounding.

### Pharmacotherapeutic Rationale for CA Capsules

Orphacol^®^ is currently the only authorized CA formulation within the EU for the treatment of 3β-HSD and 5β-reductase deficiency (EMA, 2011; Laboratoires CTRS, 2013). However, the medicine is not accessible for Dutch patients because the authorization holder has not applied for reimbursement of this product, leading to an unmet medical need for Dutch patients with BASDs. Since the efficacy and safety of CA treatment for BASD has only been studied to a limited extent (Table 1), the Amsterdam UMC decided to initiate a clinical trial (Trial NL8630). In this trial the CA treatment dose is personalized based on safety- and efficacy parameters (Table 1).

### Pharmacy and Technical Quality Assessment

Both CDCA and CA capsules are compounded in our hospital pharmacy. The pharmacy of the Amsterdam UMC has ample experience with formulation of oral preparations (capsules and oral liquids), as a lot of paediatric patients are treated in our hospital. Compounding is performed in accordance with the Dutch law. The Amsterdam UMC pharmacy holds a GMP license for manufacturing of investigational medicinal products (packaging, labelling and manufacturing of capsules). Both CDCA and CA are formulated in hard capsules which is a validated process in our pharmacy. Capsules are manually compounded by trained personnel with apparatus suitable for 100 or 300 capsules at a time. Based on the relatively small number of patients who are treated with CDCA or CA we concluded that preparation could be performed within our pharmacy.

## Sourcing of Active Pharmaceutical Ingredient

The pharmacist is responsible for the quality of the compounded formulation and is therefore inherently responsible for the quality of the active pharmaceutical ingredient (API). This responsibility starts with the selection and auditing of suppliers and all involved parties (Figure 1B). In other words, the pharmacist needs to verify that each step in the supply chain meets the valid requirements for that specific part of the chain. . It must be ensured that the API is produced according to Good Manufacturing Practices (GMP) and that transport is according to Good Distribution Practices (GDP). For common APIs, the wholesaler has already performed the supplier selection and audit, and can often share required documentation with the pharmacist.

However, for the treatment of rare diseases, APIs are usually not readily available at wholesalers. Consequently, selection and qualification of an API supplier has to be performed by the pharmacist himself or it can be outsourced to a qualified third party.

### Sourcing of Active Pharmaceutical Ingredients CDCA and CA

For both CDCA as CA we found Dutch API suppliers with connection to a API manufacturer. The manufacturers of both API’s are located outside the EU and were recently audited on EU-GMP by an independent party. An audit report was provided which covered all critical points ensuring API production according to GMP (Table 1). Throughout the supply chain, transport was performed under GDP conditions. The suppliers and API manufacturers were approved by the pharmacy and a quality agreement was drafted with the suppliers, after which the APIs were imported.

## Quality Control of Active Pharmaceutical Ingredient

APIs must comply with the general Ph. Eur. Monograph for “*Substances for Pharmaceutical Use*” (Ph.Eur., 2018b) (Figure 1C). Knowledge on the API synthesis route is required to determine how QC of the API should be performed. For setting up adequate QC analysis, information on the use of heavy metals, solvents and/or reagents during the API production process is required. Knowledge on the stability and decomposition profile of the API is also required as storage conditions can affect the quality of the API. The Drug Master File (DMF) of the manufacturer is the main source for information on API production, specifications, QC and stability. Eventually, the content and purity of the API should be guaranteed and possible harmful substances must be within the permitted limits or absent. The International Council for Harmonisation of Technical Requirements for Pharmaceuticals for Human Use (ICH) has drawn up specific guidelines for related substances, metal impurities, residual solvents and microbiological quality (ICH, 2006; ICH, 2017; ICH, 2019; ICH, 2021)

When, for whatever reason, the synthesis route of the API is unknown, a broad pharmaceutical QC analysis should be set up according the general substance monographs of the Ph. Eur. and ICH guidelines, and must be in line with appropriate quality risk management principles (Ph.Eur., 2018b; ICH, 2015) If appropriate QC of the API cannot be guaranteed, then the assessment must be made that one should refrain from using the API (Figure 1B).

### Quality Control of Active Pharmaceutical Ingredients CDCA and CA

Since the CDCA and CA APIs were imported from outside the EU, we subcontracted a certified EU laboratory to perform a full independent QC analysis of both APIs according to the GMP guidelines (European Commision, 2014). We requested the DMF from the manufacturers. The manufacturer of CDCA was willing to share the DMF after signing a confidentiality agreement. The manufacturer of CA was not willing to share the complete DMF, but provided information on the synthesis route and stability.

CDCA was manufactured according to the synthesis route described in the Ph. Eur. and QC could be performed according to the specific Ph. Eur. substance monograph for CDCA (Ph.Eur., 2017a) and the general monograph “*Substances for Pharmaceutical Use*” and ICH-GMP guidelines (Ph.Eur., 2018b, ICH, 2021; ICH, 2019; ICH, 2015; ICH, 2006; ICH, 2017).

A specific Ph. Eur. substance monograph was not available for CA. Therefore, CA API specifications were in accordance to the information obtained from the manufacturer, the Ph. Eur. substance monographs of its related compounds and the general monograph “*Substances for Pharmaceutical Use*” and ICH-GMP guidelines (Ph.Eur., 2018b, ICH, 2021; ICH, 2019; ICH, 2015; ICH, 2006; ICH, 2017) (Table 1).

Several batches of CDCA and CA API underwent QC analysis and met the specifications.

## Formulation Development

In some situations it is possible to adapt an authorized medicine for a pharmacy compounded formulation. For patients with rare diseases however, an authorized medicine is often unavailable and the pharmacy has to use starting materials. Either way, the chosen formulation determines the quality specifications and QC analysis of the medicinal product. The Dutch Pharmacists’ Association (KNMP) published a formulary handbook (also available in English) (Bouwman-Boer et al., 2015a) for the formulation, compounding and QC analysis of some of the most common dosage forms (i.e. orals, rectals, parentals, dermatics, oculars and nasals).

### Formulation Development of CDCA and CA Capsules

Fairly simple commercial immediate release capsule formulations exist for CDCA and CA (Table 1), so we investigated capsulation of the API’s in hard capsules in our pharmacy. During the first steps of formulation development, it was obvious that both API powders had poor flow properties. The addition of colloidal silica (0.5 and 1.0% for CDCA and CA capsules respectively) resulted in major improvement in flowability. Lactose monohydrate was used as a bulking agent because of its inert characteristics. For most capsule strengths (except for the 250 mg capsule), the addition of a lactose monohydrate was required (Table 1).

There was a period during which no adequate CDCA API was available. During this time Dutch patients could temporarily get the commercial CDCA Leadiant^®^ 250 mg. The Dutch Health insurance companies reimbursed the authorized medicine temporarily to ensure that all adult CTX patients could continue their treatment. For ten pediatric CTX patients however, deviant capsule strengths were required and a pharmacy compounded formulation was still necessary. As no adequate API was available at the time, the commercial product was diluted with filler (lactose monohydrate) and subsequently transferred into smaller capsules which were more suitable for the children.

## Quality Control of Medicinal Product

The QC of a medicinal product consist of three aspects: product validation, process validation and QC analysis (Figure 1D). To set the quality parameters of the medicinal product, any interaction between API, excipients, and production steps has to be assessed during the product development (Bouwman-Boer et al., 2015b). Limits need to be specified for critical production parameters (e.g., blending, apparatus, weighing, filtration, filling, volume control) and quality parameters (e.g., appearance, content, pH, (microbiological) impurities, uniformity) to ensure the quality of the medicinal product (Bouwman-Boer et al., 2015b). During the product validation the impact of critical parameters on product quality has to be studied and it should be assessed whether the specified limits are set accordingly (Bouwman-Boer et al., 2015c).

A validated process is required to ensure that the production process is robust enough to manage critical production steps and to ensure that the medicinal product is consistently produced with the intended quality (Bouwman-Boer et al., 2015d). Whereas product validation is location independent, process validation is facility-based and so the following parameters should be taken into account: facility properties, equipment, utilities, automated systems, cleaning methods, analytical methods, and training of personnel (Bouwman-Boer et al., 2015c; Bouwman-Boer et al., 2015d).

Once all critical production- and quality parameters have been determined, a QC analysis protocol for the medicinal product can be drafted. For the most common dosage forms, the primary quality parameters and specifications are described in the Ph. Eur. There is a general monograph “*Pharmaceutical Preparations*” (Ph.Eur., 2013) and for the most common dosage forms a specific monograph exists. In principle, the medicinal product should comply with both monographs. There is a practical guideline for pharmacists on how to perform QC analysis on medicinal products (Bouwman-Boer et al., 2015a).

### Quality Control of CDCA and CA Capsules

Specifications for the CDCA and CA capsules have been set according to general Ph. Eur. monographs “*Pharmaceutical Preparations*” (Ph.Eur., 2013) and “*Ca*p*sules*” (Ph.Eur., 2018a). For both products we performed a product validation (Table 1). In our pharmacy a product validation is required when more than 50 batches are prepared in a year and also for products used in a clinical study. As CDCA capsules are compounded for individual patients, batch sizes are small and QC is limited to non-destructive analysis (Table 1). When the capsules comply to the specifications mentioned in Table 1, they also comply to Ph. Eur. monograph Capsules (Specifications for the CDCA and CA capsules have been set according to general Ph. Eur. monographs “*Pharmaceutical Preparations*” (Ph.Eur., 2013) and “*Ca*p*sules*” (Ph.Eur., 2018a). For both products we performed a product validation (Table 1).

For the CA capsules the product specifications are closely similar to those set for the CDCA capsules. However, as CA treatment is given in a clinical study and is kept on stock, each batch is subject to independent QC analysis (European Commision, 2014) (Table 1). QC analysis is the same for each batch and not batch size dependent. At this moment stability testing of both products is ongoing. Preliminary shelf life has been set for 6 and 3 months respectively for the pharmacy compounded CDCA and CA capsules.

## Discussion

Pharmacy compounded medicines can provide an essential and safe option for the treatment of patients with rare diseases when authorized orphan medicines are unavailable or unsuitable for the intended use. The flowchart we developed (Figure 1) provides practical guidance for the development of pharmacy compounded formulation.

Recent evaluation of the “Orphan Regulation” (Regulation 141/2000) and the “Paediatric Regulation” (Regulation, 2006)—both adopted by the European Commission in 2000 and 2006 respectively to stimulate the development of orphan medicines and paediatric formulations—showed that the accessibility of orphan medicines varies considerably across EU member states (European Commission, 2020). Differences in national prices, reimbursement systems, prescribing behaviour, and pharmaceutical companies’ strategies, are indicated as the main causes for unequal accessibility within the EU. This is also the case for CDCA and CA treatment in the Netherlands. Reimbursement for CDCA treatment was stopped when the price increased 500 fold (Dutch National Health Care Institute, 2018). For CA, the authorization holder has not applied CA for reimbursement in the Netherlands. Pharmacy compounded CDCA and CA capsules provide a suitable and financially feasible alternative for patients. Next to this, pharmacy compounded formulations allow for more personalized treatment. In particular in the case of CA treatment, the pharmacy compounded formulations provided more flexibility in dosages, facilitating easy dose adjustments based on serum biochemical profile, liver transaminases levels and side effects (Table 1).

Pharmacy compounding especially provides improved treatment accessibility for children with rare diseases. Neither the “Orphan Regulation” nor the “Paediatric Regulation” has led to a boost in the development of innovative medicines for children with rare diseases (European Commision, 2020). Moreover, authorized medicines often have to be adjusted for children as most dosage strengths have been standardized for adults. CDCA Leadiant^®^ for example, is authorized for the treatment of CTX patients from 1 month old, with a starting dose of 5 mg/kg/day divided over three doses (Leadiant, 2017). However, CDCA Leadiant^®^ is only available in 250 mg capsules (Leadiant, 2017) and is therefore unsuitable for younger patients. As clinical studies show, early start of CDCA treatment is critical as it can improve disease prognosis and can reverse—or prevent—the development of neurological symptoms (Salen and Steiner, 2017; Stelten et al., 2019; Verrips et al., 2020). As rare diseases are often diagnosed in childhood, it is extremely important that peaditric formulations are made available.

There are several challenges in pharmacy compounding for rare diseases. It is increasingly difficult to source APIs that comply with EU laws and regulations as manufacturing of QPI; s has shifted to Asia, the number of API suppliers is limited and supply chains are often protected by market authorisation holders. In our opinion, pharmaceutical companies should recognize pharmacy compounded medicine as an addition to their products and not as a thread. Especially when dosage forms are needed that are not commercially available. We therefore encourage sharing of information between pharmacists, health care providers, pharmaceutical companies, health insurers and governments. Furthermore, pharmacists involved in compounding for patients with a rare disease should share information and knowledge on their developed formulations. Especially because there is no harmonized regulation worldwide for pharmacy preparations. Moreover, the costs of pharmacy compounded products will increase as sourcing of API, development of products and quality control, maintaining facilities takes more and more time and effort. Sharing this information across borders can help improve the inequality in accessibility of orphan medicines between countries.

All involved parties in the health care sector have the responsibility to keep healthcare accessible and affordable, and should support initiatives that contribute to that cause. In the Netherlands we see positive developments in the recognition of pharmacy compounded formulations in pharmacotherapeutic treatments for patients. This is important, because less pharmacists are interested in compounding as the revenues don’t compensate the necessary investments. It should be acknowledged that pharmacy compounded medication is an essential part of the treatment of patients with a rare disease.

## Data Availability

The original contributions presented in the study are included in the article/supplementary material, further inquiries can be directed to the corresponding author.
